# Report on Disenfectants

**Published:** 1866-07

**Authors:** 


REPORT ON DISINFECTANTS.

The first paper called up was the Report on Disinfectants, which was read by Dr. E. M. Hunt, of New Jersey, chairman of the committee. On motion to refer it to the Committee on Publication, it was brought under discussion by Dr. T. Antisell, who, though he considered the report in many respects an able one, did not regard it sufficiently elaborate, particularly in the special utility of dry heat for the destruction of fungous organisms resulting from the exposed excreta of persons sick of the cholera. While he regarded the most scrupulous cleanliness and care in the removal as speedily as possible, of all accumulations of whatever kind from such persons; yet he thought that here disinfectants had their chief application, and that if means could be divised for the thorough application of heat to all such accumulations, the poisonous quality for the diffusion of the epidemic would be destroyed. Of ozone and other disinfectants dwelt upon by the committee, he thought them of much less importance; and that they occupied too large a space in the report, to the exclusion of that which he considered so much more useful.

Drs. Green, C. A. Lee, E. R. Squibb, Jas. Hibberd took issue with Dr. Antisell as to the province of the committee in the report, regarding it in the main as sufficiently elaborate, and more strictly in accordance with what reports for the Transactions of the Association should be, than if it went into collateral subjects.

Drs. Hunt and Bell defended the report on the ground that it was necessarily a chief effort on the part of the committee to present a resumé of such facts as had from time to time been brought to notice in the history of disinfectants; and that it would not only be out of the province of the committee, but quite impossible, to report upon the special indications for the application of disinfectants to the various supposed causes of disease, such as the one presented by Dr. Antisell.

The motion to refer the paper to the Committee on Publication was then put, and the paper was so referred.

On motion, it was voted that Dr. Antisell be requested to prepare a paper for the next Session of the Association, *On the Causes of Epidemics.*

